# Empyema

**Published:** 1874-04-01

**Authors:** J. B. Rood

**Affiliations:** Lemont, Cook County, Illinois


### EMPYEMA.

By J. B. Rood, Lemont, Cook County, Illinois.

THE patient, Russell Cleveland, aged seventeen years, was taken with measles in June, 1872, which was followed with hydrothorax. July 28th, 1872, Dr. W. P. Pierce was called in consultation, and found the following physical signs: Dullness over the left lung, and bulging out between the intercostal spaces between the second, third, fourth, and fifth ribs; pulse, 140, and seventy-five respirations per minute. The heart was pressed over to the right side. He was suffering from extreme anxiety, and suffocative symptoms, speaking with difficulty, and in a short, jerking voice. His countenance was blue; the extremities were cold; the respiratory murmur was everywhere absent on the left side. Dr. Pierce suggested that paracentesis be performed; but the patient and parents refused; but as the symptoms grew worse, they consented, and on July 30th, two days later, he performed paracentesis.

The chest was opened on the left side, three inches back from the left nipple, between the fourth and fifth ribs. About three pints of purulent fluid flowed out. He was immediately relieved, and was enabled to sleep. The wound was kept open, and washed out each day with a weak solution of carbolic acid. It continued to discharge from eight to ten ounces each day. His strength returned slowly, and during this last summer he has ridden out nearly every day and was accustomed to hunt more or less during the months of July and August.

Four weeks ago the opening became closed, and for two weeks he seemed to feel much better, until March 1st, when he was taken much worse, which continued until March 8th, when he died.

The next day Dr. J. W. Comes and myself made a *post-mortem*. We found, in opening the chest, that the left thorax was filled with purulent fluid, at least two quarts. The lung-substance on that side was entirely gone; not anything left that would indicate that there was ever a lung on that side. The heart was on the right side; apex was between the fifth and sixth ribs, four inches from the sternum. The left auricle was directly under the center of the sternum, between the second and third ribs. The pericardium was adherent to the sternum, and was filled with pus. The left ventricles and auricles contained a white, glutinous substance, which I should think would weigh about an ounce and a half. The walls of the heart were very much thinned.

There was nothing abnormal in the condition of the right lung. The patient's pulse for the last year has been 112 most of the time. He was kept on tonics and cod-liver oil all the time.

